# Determinants of Mortality in Patients with Nosocomial *Acinetobacter baumannii* Bacteremia in Southwest China: A Five-Year Case-Control Study

**DOI:** 10.1155/2018/3150965

**Published:** 2018-06-03

**Authors:** Shuangshuang Yang, Jide Sun, Xianan Wu, Liping Zhang

**Affiliations:** Department of Laboratory Medicine, The First Affiliated Hospital of Chongqing Medical University, No. 1, Youyi Road, Yuzhong District, Chongqing 400016, China

## Abstract

**Purpose:**

This study was aimed to identify the determinants of in-hospital mortality in *Acinetobacter baumannii* (*A. baumannii*) bacteremia and to assess impact of carbapenem resistance on mortality.

**Methods:**

A five-year case-control study was conducted from January 2011 to December 2015 in a tertiary teaching hospital with 3200 beds, Southwest China. Clinical outcomes and potential determinants of mortality in patients with nosocomial *A. baumannii* bacteremia and carbapenem-resistant *A. baumannii* (CRAB) bacteremia were evaluated using Cox and logistic regression analyses.

**Results:**

A total of 118 patients with nosocomial *A. baumannii* bacteremia were included. Seventy-one percent (84/118) of them had carbapenem-resistant *A. baumannii* (CRAB) bacteremia. The in-hospital mortality of nosocomial *A. baumannii* bacteremia was 21.2%, and the attributable in-hospital mortality rate due to CRAB was 21.5%. Significant difference of 30-day in-hospital mortality in the Kaplan–Meier curves was found between CRAB and CSAB groups (log-rank test, *P*=0.025). The Cox regression analysis showed that patients with CRAB bacteremia had 2.72 times higher risk for 30-day in-hospital mortality than did those with carbapenem-susceptible *A. baumannii* (CSAB) bacteremia (95% confidence intervals (CIs) 1.14–6.61, *P*=0.016). The logistic regression analysis reported that mechanical ventilation and respiratory tract as origin of bacteremia were independent predictors of mortality among patients with nosocomial *A. baumannii* bacteremia and CRAB bacteremia, while high APACHE II score on the day of bacteremia and multiple organ dysfunction syndromes (MODS) during hospitalization were independent predictors of mortality among patients with nosocomial *A. baumannii* bacteremia but not CRAB bacteremia.

**Conclusion:**

It was the severity of illness (high APACHE II score and MODS) not carbapenem resistance that highlighted the mortality of patients with nosocomial *A. baumannii* bacteremia. The impact of mechanical ventilation on mortality suggested that respiratory dysfunction might prime the poor outcome. Protection of respiratory function during the progression of nosocomial *A. baumannii* bacteremia should be given more importance. Early identification and intervention of patients with nosocomial *A. baumannii* bacteremia in critical ill conditions were advocated.

## 1. Introduction

The past two decades have witnessed a dramatic rise in the incidence of nosocomial infections by *Acinetobacter baumannii* (*A. baumannii*) with a worrisome morbidity and mortality [1]. Its global spread is generally attributed to the inherent or acquired ability of environment resilience, transmission, and resistance to broad-spectrum antimicrobials.

As a major nosocomial pathogen, carbapenem-resistant *A. baumannii* (CRAB) is of particular concern. Recent surveillance has reported its high prevalence and causative agent in ventilation-associated pneumonia (VAP), bloodstream infection, surgical site infection, postsurgical meningitis, and abdominal infection [2]. Bloodstream infection caused by CRAB has been a more tremendous clinical challenge in consideration of the lack of effective antibiotic agents and high mortality [3–6]. Despite colistin and tigecycline as the last resorts, the optimal therapeutic regimen of CRAB bacteremia has not been established, and the unavailability of new antibiotic pipeline in developing countries definitely restricts clinical therapeutic options and facilitates its dissemination. Therefore, surveillance of CRAB bacteremia and selection of patients for CRAB empirical coverage have been considered more essential. Numerous data from different regions have elucidated risk factors for nosocomial colonization or infection of CRAB, whereas impact of nosocomial bacteremia with CRAB has not been well documented, especially in China, and there is ongoing controversy as to whether carbapenem resistance determines a higher mortality of *A. baumannii* bacteremia.

Accordingly, the aim of this study was to identify the determinants of mortality for nosocomial *A. baumannii* bacteremia and further to illustrate the potential impact of carbapenem resistance on in-hospital mortality in this setting.

## 2. Patients and Methods

A five-year study of nosocomial *A. baumannii* bacteremia was conducted from January 2011 to December 2015 in the First Affiliated Hospital of Chongqing Medical University, which is the surveillance center of antimicrobial resistance in Chongqing, Southwest China. Approval was obtained from the Ethics Committee of the First Affiliated Hospital of Chongqing Medical University.

### 2.1. Study Design

To meet the objectives, a case-control study design was applied. Nonduplicated hospitalized patients with *A. baumannii* bacteremia were enrolled. Patients with incomplete medical data, younger than 18 years old, or with multiple microbial infections were excluded. The determinants of mortality of patients with nosocomial *A. baumannii* bacteremia were studied by comparing demographics, clinical characteristics, and clinical interventions between nonsurvival cases and survival controls. Then nonsurvival cases and survival controls with nosocomial CRAB bacteremia were further compared, and predictors of mortality of patients with nosocomial CRAB bacteremia were determined.

### 2.2. Microbiological Methods

Blood specimens were obtained by trained nurses and cultured in BD FX200 blood culture systems. Microbiological identification and antibiotic susceptibility testing were performed by the VITEK 2 compact (bioMérieux, France). Carbapenem susceptibility was interpreted by the minimal inhibitory concentration (MIC) breakpoints of Clinical and Laboratory Standards Institute (CLSI) M100-S26.

### 2.3. Definitions

Nosocomial *A. baumannii* bacteremia was defined as patients with positive blood culture only for *A. baumannii* and with clinical signs or symptoms of infection at least 48 hours after hospitalization [5].

Carbapenem-susceptible *A. baumannii* (CSAB) was defined as MIC ≤ 2 *μ*g/mL for imipenem or meropenem, and carbapenem-resistant *A. baumannii* (CRAB) was defined as MIC ≥ 8 *μ*g/mL for imipenem or meropenem.

Multidurg resistance was defined as resistance to no less than 3 of the following antibiotic agents: antipseudomonal cephalosporins, antipseudomonal carbapenems, ampicillin/sulbactam, fluoroquinolones, and aminoglycosides [7].

Previous use of antibiotic agents was considered if antibiotic therapy had been adopted for more than 3 consecutive days within 30 days prior to the collection of the first blood specimen for culture [8].

Administration of at least one effective antimicrobial agent for 48 h, within 5 days of the onset of bacteremia, is defined as adequate empirical antimicrobial therapy, and if the causative pathogen was susceptible to it in vitro, then it is defined as an effective antimicrobial agent [9].

### 2.3. Data Collection

Demographics, clinical characteristics, and clinical outcomes were retrieved from electronic database of the hospital information system (HIS), and microbiological characteristics were retrieved from the laboratory information system (LIS). Clinical characteristics were comorbidity, Acute Physiology and Chronic Health Evaluation (APACHE) II score on admission, APACHE II score on the day of bacteremia, multiple organ dysfunction syndromes (MODS), hospital wards, origin of bacteremia, previous antibiotic agent use, the duration of antibiotic agent exposure before the onset of *A. baumannii* bacteremia, and invasive procedures (abdominal or thoracic drainages, central venous catheterization, mechanical ventilation, nasogastric tube, and urinary catheterization). Clinical outcomes were reported with death or survival by clinicians in the medical records, and only in-hospital mortality was captured.

### 2.4. Statistical Analysis

Univariate analysis was performed to identify the determinants of mortality of *A. baumannii* bacteremia and CRAB bacteremia. Chi-square or Fisher's exact test was used to compare categorical variables. Student's *t*-test or the Mann–Whitney *U* test was used to compare continuous variables. Variables with a *P* value < 0.05 in the univariate test were included in a multivariate logistic remodel. The odds ratio (OR) and its 95% confidence interval (CI) were calculated to evaluate the strength of any association. Kaplan–Merier curves were compared by the log-rank test. Hazard ratio was calculated by the Cox regression analysis. A two-tailed *P* value < 0.05 was considered as statistically significant. SPSS v.21.0 (SPSS Inc., Chicago, IL) was used to perform all of the statistical calculations.

## 3. Results

### 3.1. Demographic, Clinical, and Microbiological Characteristics of Nosocomial *A. baumannii* Bacteremia

During the investigation period, 136 nonduplicate patients were retrieved with nosocomial *A. baumannii* bacteremia, and due to exclusion criteria, 18 of them were excluded. A total of 118 patients were enrolled from 19 wards in the different parts of this hospital. The mean age of this study population was 58.2 years. Sixty-seven percentage (79/118) of them were male. Sixty-six percentage (78/118) of patients suffered from underlying comorbidities. Hypertension was the most common underlying disease, presenting in 38.1% of the patients. Among the 118 patients, 84 patients had yielded CRAB, and the overall incidence of CRAB was 71.2% (84/118). ICU was the main source of both nosocomial *A. baumannii* bacteremia (61/118, 51.7%) and CRAB bacteremia (55/61, 90.2%), followed by neurosurgery department and hepatobiliary surgery department. As to subareas of ICU, general ICU contributed a large percentage of CRAB bacteremia cases (33/61, 54.1%), followed by respiratory ICU (15/61, 24.6%).

Of 118 *A. baumannii* isolates, 77.8% were multidrug resistant and 71.2% were CRAB. Similar but high resistant rates (77.8%) to ampicillin/sulbactam, piperacillin, piperacillin/tazobactam, ceftazidime, ceftriaxone, and ciprofloxacin were observed, whereas those to levofloxacin and sulfamethoxazole were no more than 40%. In comparison, relatively low resistance rates to minocycline and cefoperazone/sulbactam were found with resistance rates of 6.8% and 22.2%, respectively. As to the 84 CRAB strains, all of them were multidrug resistant and resistant to ampicillin/sulbactam, piperacillin, piperacillin/tazobactam, ceftazidime, ceftriaxone, cefepime, and ciprofloxacin. Resistance rates of CRAB strains to minocycline and cefoperazone/sulbactam were 8.6% and 28.6%, respectively.

### 3.2. Clinical Outcomes of Nosocomial *A. baumannii* Bacteremia

**Figure 1:**
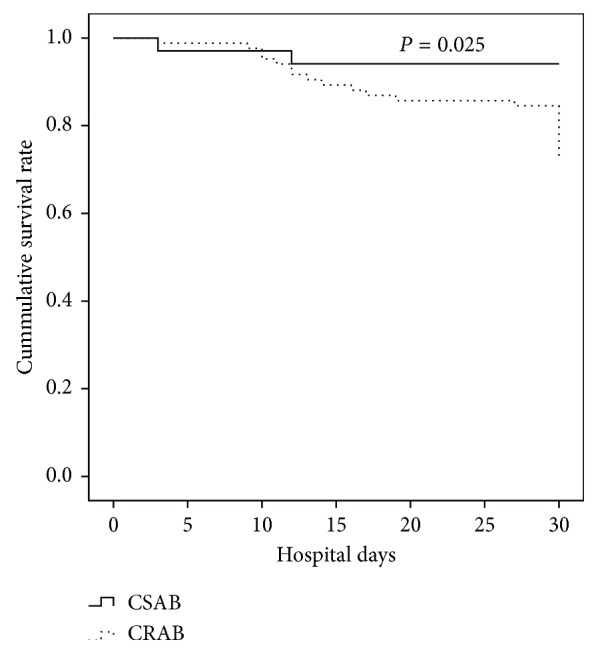
Risk of 30-day in-hospital mortality comparing patients with CRAB bacteremia and patients with CSAB bacteremia by the log-rank test.

Totally 25 of 118 patients with nosocomial *A. baumannii* bacteremia died during the study period, yielding an in-hospital mortality rate of 21.2%. The crude mortality rate of patients with CRAB bacteremia was 27.4% (23/84), while that of patients with CSAB bacteremia was 5.9% (2/34). Significant difference was observed between patients with CRAB and CSAB bacteremia in mortality rate (*P*=0.011). The attributable mortality rate due to CRAB was 21.5%, calculated by the subtraction of the crude mortality rate of CSAB patients from that of CRAB patients [10]. Overall, the 30-day in-hospital mortality rate of patients with nosocomial *A. baumannii* bacteremia was 12.7% (15/118). The 30-day in-hospital mortality rate of CRAB patients was 15.5% (13/84), while that of CSAB patients was 5.9% (2/34). The log-rank test found a significant difference in the Kaplan–Meier curves of 30-day in-hospital mortality between CRAB and CSAB patients (*P*=0.025). Furthermore, Cox regression analysis found that patients with CRAB bacteremia suffered 2.72 times higher risk of mortality than did patients with CSAB bacteremia in 30-day hospitalization (hazard ratio (HR) = 2.72; 95% CI 1.14–6.61; *P*=0.016; Figure 1).

### 3.3. Predictors for the Mortality of Nosocomial *A. baumannii* Bacteremia

Although our results have indicated that patients with CRAB bacteremia were vulnerable to death, it is arbitrary to deduce that carbapenem resistance is the predictor for the mortality of *A. baumannii* bacteremia in nosocomial scenarios. Therefore, we further evaluated the determinants of the mortality of nosocomial *A. baumannii* bacteremia (Table 1). Twenty-five nonsurvivors and 93 survivors were included. Univariate analysis found that nonsurvivors were more likely to suffer critical conditions than survivors, as indicated by an increased APACHE II score on admission, APACHE II score on the day of bacteremia, and MODS (*P*=0.01,  *P*=0.001,  and *P*=0.005). As expected, APACHE II score on admission, APACHE II score on the day of bacteremia, carbapenem resistance, central venous catheterization, mechanical ventilation, MODS, ICU stay before the bacteremia day, and respiratory tract as the origin of bacteremia were associated with the mortality of patients with nosocomial *A. baumannii* bacteremia, while adequate empirical antibiotic therapy was associated with their survival. By logistic regression analysis, high APACHE II score on the day of bacteremia, MODS, mechanical ventilation, and respiratory tract as the origin of bacteremia were identified as independent predictors for the mortality of nosocomial *A. baumannii* bacteremia (Table 2). Interestingly, carbapenem resistance was not significantly associated with the mortality of nosocomial *A. baumannii* bacteremia.

### 3.4. Carbapenem Resistance and the Mortality of Nosocomial *A. baumannii* Bacteremia

Since carbapenem resistance did not significantly contribute to the mortality of nosocomial *A. baumannii* bacteremia, the determinants of mortality of nosocomial CRAB bacteremia was further studied. Twenty-three nonsurvivors and 61 survivors with CRAB bacteremia were compared by univariate analysis.  Table 3 shows univariate analysis of risk factors for the mortality of nosocomial CRAB bacteremia. No significant difference was found between two groups in terms of male gender, age, and comorbidities. MODS, mechanical ventilation, and respiratory tract as the origin of bacteremia were found significantly associated with the mortality of nosocomial CRAB bacteremia. Logistic regression analysis also found that mechanical ventilation (OR, 7.39; 95% CI, 1.39–39.17; *P*=0.019) and respiratory tract as the origin of bacteremia (OR, 7.41; 95% CI, 2.20–24.89; *P*=0.001) were strong independent risk factors for the mortality of CRAB bacteremia.

## 4. Discussion

Limited therapeutic alternatives, a high morbidity, and mortality have rendered surveillance of CRAB a top priority. High prevalence of CRAB (71.2%) was observed in this study, especially in ICU, which is consistent with latest investigations from tertiary teaching hospitals of China, which had observed a steep rise in the incidence of CRAB bacteremia with high carbapenem resistance rates from 62.1% to 91% [11–13]. Furthermore, China's surveillance of antimicrobial resistance program has detected extensively drug-resistant *A. baumannii* (XDRAB), the most frequently reported Gram-negative bacteria isolated from bloodstream, and 97.4% of XDRAB were resistant to imipenem [14]. Similar to other studies [15, 16], all the CRAB isolates were multidrug resistant but retained their susceptibility to minocycline and cefoperazone/sulbactam, suggesting the combination of antibiotic therapy of minocycline and/or cefoperazone/sulbactam may benefit the clearance of CRAB in this setting.

Despite high prevalence of CRAB in China, the impact of carbapenem resistance on *A. baumannii* bacteremia has not been well documented. In this study, the crude and 30-day in-hospital mortality rate of nosocomial *A. baumannii* bacteremia were 21.2% and 12.7%, respectively, which are similar to the results of a five-year Japanese study [17], but relatively lower than other previous reports with high mortality rates from 29% to 63.5% [12, 18, 19]. This inconsistency is probably ascribed to the heterogeneity of epidemiology, the severity of diseases and the virulence factors of microbes.

Higher mortality rate was detected among the CRAB group during hospitalization, which is consistent with a four-year study of bloodstream infection concerning multidrug-resistant Gram-negative bacteria [20]. Surprisingly, after adjusting for potential confounders, no significant association was found between carbapenem resistance and increased risk of in-hospital mortality.

On the contrary, high APACHE II score on the day of bacteremia, MODS, and respiratory tract as the origin of bacteremia were independent predictors for the mortality of nosocomial *A. baumannii* bacteremia. Recently, studies focused on ICU patients have shown that it is not antibiotic resistance but the severity of the illness that highlights in-hospital mortality of nosocomial *A. baumannii* bacteremia [21–23]. These results were compatible with our findings. High APACHE II score on the day of bacteremia and MODS during hospitalization concordantly suggested critical ill conditions, which may lead to rapid death during hospitalization. Furthermore, a recent study in regard of the attributed mortality of nosocomial *A. baumannii* bacteremia by hospital acquired pneumonia (HAP) in South China found that high APACHE II score was the independent risk factor for HAP patients complicated with bloodstream infection and was associated with high mortality in nosocomial *A. baumannii* bacteremic pneumonia [24]. Accordingly, it is the severity of illness that predicts the mortality of nosocomial *A. baumannii* bacteremia in this setting.

Furthermore, this study found that mechanical ventilation increased the risk of mortality of patients with nosocomial *A. baumannii* and CRAB bacteremia, which is consistent with other studies [11, 19, 25]. Since a majority of patients in this cohort were suffered ICU admission and it has been reported that more than 75% of CRAB bacteremia were due to CRAB ventilation-associated pneumonia in ICU [9, 24], it seems reasonable to expect mechanical ventilation as a pivotal predicator of patients to acquire CRAB bacteremia. However, mechanical ventilation did not predict acquisition but mortality in this cohort. The severity of illness entailed the intervention of mechanical ventilation, but the consequent ventilator-induced lung injury and ventilation associated pneumonia may aggravate the ill conditions and contribute to the mortality [4, 24, 26]. Moreover, this result also suggests that respiratory failure, a critical ill status, is probable to be a hint of poor outcomes of patients with nosocomial *A. baumannii* bacteremia regardless of CRAB or CSAB. Thus, noninvasive techniques for respiratory support, alternative lung-protective ventilation to prevent ventilator-induced lung injury, and personalization of mechanical ventilation based on individual physiological characteristics and responses to therapy are in urgent need and probably improve outcomes [26].

Interestingly, adequate antibiotic empirical therapy was not associated with mortality of patients with nosocomial *A. baumannii* and CRAB bacteremia in the current study. Although carbapenem resistance itself increased three- to fivefold risk for receiving inappropriate carbapenem antibiotic therapy [9, 27] and previous studies have found that inappropriate empirical antibiotic therapy increases the mortality of patients with CRAB bacteremia and bacteremic pneumonia [9, 27, 28], our study failed to find that adequate empirical therapy benefitted the survival of patients with nosocomial *A. baumannii* or CRAB bacteremia. The controversy of the impact of appropriate/inappropriate empirical therapy on mortality of *A. baumannii* bacteremia is ongoing. Several studies have reported that the appropriateness of antibiotic therapy does not affect early mortality rate, and inappropriate antibiotic therapy is not associated with short-term mortality [19, 29]. Further study illustrated that appropriate antimicrobial therapy did not significantly benefit patients with APACHE II no more than 25 [30]. These results were consistent with our observation, which found an average APACHE II score of 22.2 in nonsurvival patients and that of 18.0 in survival patients with *A. baumannii* bacteremia. However, it should be noticed that less than 50% of patients received appropriate empirical antibiotic therapy in this present study, due to the unavailability of first-line antibiotic agents: colistin and tigecycline. Two studies concerning about bloodstream infection caused by extensively drug-resistant *A. baumannii* in critical ill patients found that it was the ineffective management of such infection that increased the risk of death and colistin-vancomycin coadministration was a predictor of a good prognosis [31, 32]. Accordingly, we deduced that the beneficial impact of appropriate empirical antibiotic therapy on clinical outcome might be underestimated in this study.

Since this present study has identified that the severity of illness predicts the fate of patients with nosocomial *A. baumannii* bacteremia, rapid identification of patients endangered by nosocomial *A. baumannii* bacteremia deserves top priorities. MALDI-TOF MS in direct blood sample detection may contribute to improve the early management of *A. baumannii* bacteremia. A recent pre-post, quasi-experimental study witnessed reduced length of time for the clearance of Gram-negative rods in bloodstream infection by the combination of MALDI-TOF MS and real-time antimicrobial stewardship intervention on time [33]. Recently, CarbAcineto NP test (CANP) has been reported with a high sensitivity to detecting OXA-type carbapenemases, which are more frequently identified in carbapenem-resistant *Acinetobacter* spp. [34]. Its combination with MALDI-TOF MS in direct blood sample detection may contribute to improve the early management of CRAB bacteremia. Based on molecular techniques, the FilmArray System Blood Culture Identification (BCID) panel and novel bioluminescence-based phenotypic method have been found helpful for clinicians to identify patients suspected for *A. baumannii* and CRAB bacteremia in a timely manner [35, 36]. Moreover, prevention of its acquisition and progression should be emphasized. Recent overview of clinical pathogenesis of *A. baumannii* infection found that host fate during *A. baumannii* infection was determined by two stages, which underscored the balancing of host immunological defense and bacteria offense [2]. Our previous study has reported that mucosal immunization with *A. baumannii* outer membrane protein A (OmpA) is a novel, noninvasive vaccine approach to protect mice against multidrug-resistant *A. baumannii* infections and suggests the development of OmpA as a promising protein vaccine component against multidrug-resistant *A. baumannii* in patients, considering that OmpA is immunogenic in humans [37]. A latest study in Italy has further found the potential of immunoglobulin M (IgM) in protecting patients with septic shock from deaths [6]. Further researches will shed light on the pathogenesis of *A. baumannii* infection in host with comorbidity.

Some limitations of this study are noteworthy. Firstly, as a retrospective study, possible risk or causative factors, such as potential immunocompromised status and confounding complications, were unmeasured. Secondly, molecular mechanism analysis of carbapenem resistance, especially the detection of sequence types and carbapenemase genes, was not conducted. Nevertheless, our previous two-year study of carbapenem-resistant *A. baumannii* (CRAB) had reported the predominance of sequence type (ST) 75 and ST137, both of which are attributable to clonal complex (CC) 92, and the most common carbapenemase genes were *bla*OXA-23 and *bla*OXA-51 [38]. These results are compatible with a national multicenter study in China, which found that the most widely distributed CC of CRAB isolates was *bla*OXA-23-like-producing and predominantly CC92 [39]. Furthermore, a recent study focusing on bacteremic pneumonia caused by *A. baumannii* in ICUs of South China also verified the predominance of CC92 [24]. So, it is reasonable to suppose that CC92 may be the predominant genotype and *bla*OXA-23 may be the most prevalent carbapenemase of CRAB isolates in this setting. Thirdly, single-center findings restricted their applicabilities to other geographical settings.

## 5. Conclusions

In summary, the results of the present investigation suggest high prevalence of CRAB in nosocomial bloodstream infection, especially in ICU. High in-hospital mortality rate of nosocomial *A. baumannii* bacteremia, especially among patients with CRAB, was observed, while carbapenem resistance did not predict in-hospital mortality. The severity of the illness (MODS and high APACHE II score), mechanical ventilation, and respiratory tract as the origin of bacteremia were determinants of in-hospital mortality of nosocomial *A. baumannii* bacteremia, while as to nosocomial CRAB bacteremia, mechanical ventilation and respiratory tract as the origin of bacteremia were risk factors for in-hospital mortality. The role of mechanical ventilation in mortality was highlighted, which suggests respiratory dysfunction may prime the poor outcome. The beneficial impact of adequate empirical antibiotic therapy on survival cannot be concluded but may be underestimated. Early identification, consecutive assessment of the severity of the illness, and protection of respiratory function during the spring of nosocomial *A. baumannii* bacteremia should be conducted to reduce the risk of mortality of nosocomial *A. baumannii* bacteremia. Further research should emphasize pathogenetic mechanisms of *A. baumannii* bacteremia for the evaluation of novel therapy surrogates.

## Figures and Tables

**Table 1:** Univariate analysis of the clinical characteristics of survival and nonsurvival patients suffered from nosocomial bacteremia caused by *Acinetobacter baumannii*.

Characteristics	Patients^a^		*P* value	OR	95% CI
	Nonsurvivors (*n*=25)	Survivors (*n*=93)			
Male	17 (68.0)	62 (66.6)	1.000	1.06	0.41–2.73
Age (median (IQR)) (years)	68 (58, 75)	60 (44.5, 70)	0.104	—	—
Carbapenem resistance	24 (96.0)	60 (64.5)	**0.001**	**13.20**	**1.71–102.17**
Source					
Abdominal route	0 (0)	4 (4.3)	0.577	0.39	0.02–7.49
Cerebral spinal route	1 (4)	6 (6.45)	1.000	0.60	0.07–5.27
Central venous route	1 (4)	6 (6.45)	1.000	0.60	0.07–5.27
Primary	11 (44)	60 (64.5)	0.070	0.43	0.18–1.06
Respiratory tract	11 (44)	11 (11.8)	**0.001**	**5.86**	**2.13–16.08**
Skin/soft tissue	1 (4)	4 (4.3)	1.000	0.93	0.09–8.69
Urinary tract	0 (0)	2 (2.2)	1.000	0.72	0.03–15.44
COPD	2 (8)	2 (2.2)	0.197	3.96	0.53–29.62
Coronary heart diseases	5 (20)	12 (12.9)	0.353	1.69	0.53–5.34
Diabetes mellitus	4 (16)	24 (25.8)	0.429	0.55	0.17–1.76
Hepatic diseases	4 (16)	7 (7.5)	0.243	2.34	0.63–8.74
Hypertension	6 (24)	39 (41.9)	0.112	0.44	0.16–1.20
Renal diseases	5 (20)	9 (9.7)	0.172	2.33	0.70–7.73
Malignancy	5 (20)	10 (10.8)	0.307	2.08	0.64–6.75
MODS during hospitalization	5 (20)	2 (2.2)	**0.005**	**11.38**	**2.06–62.90**
APACHE II score on admission (mean ± SD)	21.6 ± 1.4	17.8 ± 0.6	**0.010**	—	—
APACHE II score on the day of bacteremia (mean ± SD)	22.3 ± 1.3	18.0 ± 0.6	**0.001**	—	—
ICU stay	22 (88)	62 (66.6)	**0.046**	**3.67**	**1.02–13.21**
Any antibiotic exposure prior to positive blood culture	25 (100)	82 (88.2)	0.117	7.11	0.40–125.0
Duration of previous antimicrobial exposure	11 (8, 24)	13 (6.5, 23)	0.811	—	—
Aminoglycosides	8 (32)	14 (15.1)	0.079	2.66	0.96–7.33
Antifungal azoles	8 (32)	23 (24.7)	0.454	1.43	0.55–3.75
*β*-Lactam/*β*-lactamase inhibitors	11 (44)	47 (49.5)	0.654	0.769	0.32–1.87
Cefepime	2 (8)	9 (9.7)	1.000	0.81	0.16–4.02
Carbapenems	19 (76)	52 (55.9)	0.106	2.50	0.91–6.82
Fluoroquinolones	12 (32)	26 (14.0)	0.089	2.38	0.96–5.89
Minocycline	2 (8)	7 (7.5)	1.000	1.068	0.21–5.50
Third-generation cephalosporins	7 (28)	29 (31.2)	0.812	0.86	0.32–2.28
Nitroimidazoles	6 (24)	27 (29.0)	0.803	0.77	0.28–2.14
Abdominal or thoracic drainages	9 (36)	35 (37.6)	1.000	0.93	0.37–2.34
Central venous catheterization	19 (76)	47 (50.5)	**0.025**	**3.10**	**1.14–8.46**
Mechanical ventilation	22 (88)	44 (47.3)	**0.000**	**8.17**	**2.29–29.18**
Nasogastric tube	11 (44)	30 (32.3)	0.345	1.65	0.67–4.07
Urinary catheterization	15 (60)	46 (49.5)	0.376	1.53	0.62–3.76
Adequate empirical antibiotic therapy	7 (28)	53 (57)	**0.013**	**0.29**	**0.11–0.77**

APACHE: Acute Physiology and Chronic Health Evaluation; COPD: chronic obstructive pulmonary disease; CI: confidence interval; ICU: intensive care unit; IQR: interquartile range; OR: odds ratio; SD: standard deviation; MODS: multiple organ dysfunction syndrome. ^a^Data were expressed as the number (%) of patients unless stated otherwise.

**Table 2:** Multivariate logistic analysis of predictors of in-hospital mortality among patients suffered from nosocomial bacteremia caused by *Acinetobacter baumannii*.

Predictors	aOR	95% CI	*P* value
MODS during hospitalization	10.35	1.20–89.01	**0.033**
APACHE II score on admission	1.02	0.93–1.12	0.685
APACHE II score on the day of bacteremia	1.82	1.15–2.86	**0.010**
ICU stay	0.57	0.10–3.36	0.534
Mechanical ventilation	10.13	1.76–58.23	**0.009**
Central venous catheterization	1.29	0.35–4.80	0.704
Carbapenem resistance	0.93	0.11–7.81	0.94
Respiratory tract origin	10.67	2.61–43.61	**0.001**
Adequate empirical antibiotic therapy	1.43	0.35–5.92	0.620

aOR: adjusted odds ratio; CI: confidence interval; MODS: multiple organ dysfunction syndrome; ICU: intensive care unit.

**Table 3:** Univariate analysis of the clinical characteristics of survival and nonsurvival patients suffered from nosocomial bacteremia caused by carbapenem-resistant *Acinetobacter baumannii*.

Characteristics	Patients^a^		*P* value	OR	95% CI
	Nonsurvivors	Survivors			
	(*n*=23)	(*n*=61)			
Male	15 (65.2)	43 (70.5)	0.792	0.79	0.28–2.18
Age (median (IQR)) (years)	68 (46, 76)	58 (41, 70)	0.067	—	—
Source					
Abdominal route	1 (4.3)	2 (3.3)	1.000	1.34	0.12–15.55
Cerebral spinal route	1 (4.3)	4 (6.6)	1.000	0.65	0.07–6.12
Central venous route	0 (0)	4 (6.6)	0.571	0.27	0.01–5.26
Primary	9 (39.1)	34 (55.7)	0.223	0.51	0.19–1.36
Respiratory tract	11 (47.8)	11 (18.0)	**0.011**	**4.17**	**1.46–11.87**
Skin/soft tissue	1 (4.3)	4 (6.6)	1.000	0.65	0.07–6.12
Urinary tract	0 (0)	2 (3.3)	1.000	0.51	0.02–10.96
Coronary heart diseases	5 (21.7)	9 (14.8)	0.515	1.61	0.48–5.43
Diabetes mellitus	4 (17.4)	12 (19.7)	1.000	0.86	0.25–3.00
Hypertension	6 (26.1)	26 (42.6)	0.211	0.48	0.17–1.37
Hepatic diseases	4 (17.4)	6 (9.8)	0.450	1.93	0.49–7.59
Renal diseases	5 (21.7)	6 (9.8)	0.163	2.55	0.69–9.35
Malignancy	4 (17.4)	4 (6.6)	0.206	3.00	0.68–13.18
MODS during hospitalization	4 (17.4)	2 (3.3)	**0.045**	**6.21**	**1.05–36.64**
APACHE II score on admission (mean ± SD)	21.74 ± 1.42	19.18 ± 0.71	0.116	—	—
APACHE II score on the day of bacteremia (mean ± SD)	22.39 ± 1.38	19.34 ± 0.68	0.056	—	—
ICU stay	22 (95.7)	48 (78.7)	0.098	5.96	0.73–48.47
Any antibiotic exposure prior to positive blood culture	23 (100)	60 (98.4)	1.000	1.17	0.05–29.66
Duration of previous antimicrobial exposure	11 (8, 24)	14 (9, 23)	0.811	—	—
Aminoglycosides	8 (34.8)	10 (16.4)	0.080	2.72	0.91–8.12
Antifungal azoles	7 (30.4)	18 (29.5)	1.000	1.05	0.37–2.97
*β*-Lactam/*β*-lactamase inhibitor combinations	11 (47.8)	34 (55.7)	0.517	0.73	0.28–1.91
Carbapenems	18 (78.3)	40 (65.6)	0.302	1.89	0.61–5.81
Cefepime	1 (4.3)	7 (11.5)	0.436	0.35	0.04–3.02
Fluoroquinolones	12 (52.2)	22 (16.4)	0.216	1.93	0.73–5.11
Minocycline	2 (8.7)	6 (9.8)	1.000	0.87	0.16–4.67
Nitroimidazoles	6 (26.1)	23 (37.7)	0.441	0.58	0.20–1.69
Third-generation cephalosporins	6 (26.1)	22 (36.1)	0.446	0.63	0.22–1.82
Abdominal or thoracic drainages	9 (39.1)	24 (39.3)	1.000	0.99	0.37–2.65
Central venous catheterization	18 (78.3)	37 (60.7)	0.198	2.34	0.76–7.13
Mechanical ventilation	21 (91.3)	37 (60.7)	**0.007**	**6.81**	**1.46–37.74**
Nasogastric tube	11 (47.8)	23 (37.7)	0.459	1.51	0.58–3.99
Urinary catheterization	15 (65.2)	34 (55.7)	0.468	1.49	0.55–4.03
Adequate empirical antibiotic therapy	5 (21.8)	21 (34.4)	0.30	0.53	0.17–1.62

APACHE: Acute Physiology and Chronic Health Evaluation; COPD: chronic obstructive pulmonary disease; CI: confidence interval; ICU: intensive care unit; IQR: interquartile range; OR: odds ratio; SD: standard deviation; MODS: multiple organ dysfunction syndrome. ^a^Data were expressed as the number (%) of patients unless stated otherwise.

## Data Availability

The authors confirm that the data supporting the findings of this study are available within the article.
