# Corrigendum to “Determinants of Mortality in Patients with Nosocomial *Acinetobacter baumannii* Bacteremia in Southwest China: A Five-Year Case-Control Study”

**DOI:** 10.1155/2019/2790537

**Published:** 2019-05-09

**Authors:** Shuangshuang Yang, Jide Sun, Xianan Wu, Liping Zhang

**Affiliations:** Department of Laboratory Medicine, The First Affiliated Hospital of Chongqing Medical University, No. 1, Youyi Road, Yuzhong District, Chongqing 400016, China

In the article titled “Determinants of Mortality in Patients with Nosocomial *Acinetobacter baumannii* Bacteremia in Southwest China: A Five-Year Case-Control Study” [1], there was an error in the Acknowledgments section, as the funding details of the Chongqing Science and Technology Commission Grant (cstc2016jcyjA0769) was incorrect, and the correct number is (cstc2016jcyjA0248).
